# Identifying the appropriate spatial resolution for the analysis of crime patterns

**DOI:** 10.1371/journal.pone.0218324

**Published:** 2019-06-26

**Authors:** Nick Malleson, Wouter Steenbeek, Martin A. Andresen

**Affiliations:** 1 School of Geography, University of Leeds, Leeds, United Kingdom; 2 Netherlands Institute for the Study of Crime and Law Enforcement (NSCR), Amsterdam, The Netherlands; 3 Institute for Canadian Urban Research Studies (ICURS), School of Criminology, Simon Fraser University, Burnaby, BC, Canada; University of the Aegean School of Social Sciences, GREECE

## Abstract

**Background:**

A key issue in the analysis of many spatial processes is the choice of an *appropriate scale* for the analysis. Smaller geographical units are generally preferable for the study of human phenomena because they are less likely to cause heterogeneous groups to be conflated. However, it can be harder to obtain data for small units and small-number problems can frustrate quantitative analysis. This research presents a new approach that can be used to estimate the most appropriate scale at which to aggregate point data to areas.

**Data and methods:**

The proposed method works by creating a number of regular grids with iteratively smaller cell sizes (increasing grid resolution) and estimating the *similarity* between two realisations of the point pattern at each resolution. The method is applied first to simulated point patterns and then to real publicly available crime data from the city of Vancouver, Canada. The crime types tested are residential burglary, commercial burglary, theft from vehicle and theft of bike.

**Findings:**

The results provide evidence for the size of spatial unit that is the most appropriate for the different types of crime studied. Importantly, the results are dependent on both the number of events in the data and the degree of spatial clustering, so a single ‘appropriate’ scale is not identified. The method is nevertheless useful as a means of better estimating what spatial scale might be appropriate for a particular piece of analysis.

## 1 Introduction

A key issue in the analysis of many spatial processes is the choice of an *appropriate scale* for the analysis, when only one scale of analysis is being used. This choice may be based on, or identified through, theoretical means or the research question; and in some cases there may not be a choice because data have been provided at one spatial scale. It may, however, be based on the need to identify the scale at which no further disaggregation provides new information. For many phenomena in the latter context, smaller spatial units are generally preferable because they are more likely to be homogeneous with respect to both the events under study and the population at risk, and, therefore, represent more accurately the underlying spatial pattern—this would not be the case when a measure of heterogeneity is theoretically important, however. As urban socio-demographics can vary considerable over quite small distances, large spatial units may hide or “smooth out” [1] important low-level patterns. And if the relevant social processes occur at larger spatial scales, small spatial units can be aggregated easily to larger spatial units. Recognising the importance and practical benefits of (starting with) small spatial units, recent research in many social-science fields tends towards ‘micro places’. This is especially true for crime science research, which is the subject of this paper.

Furthermore, crime and place research has shown that the micro-place accounts for the majority of the variability in spatial patterns of crime. Considering the micro-level (e.g. street segments), the meso-level (larger areas approximately the size of one square kilometre, e.g. neighbourhoods or census tracts), and macro-level geographic areas that are larger than neighbourhoods but still regions within a city, researchers have found that 60 percent, or more, of spatial variation can be accounted for using micro-places [2–4]. Larger areal units still matter, but not as much as the micro-place. However, working at small spatial units poses three particular problems. Firstly, data for small spatial units are harder and more expensive to obtain. Data are more likely to be personally disclosive or commercially sensitive so have greater restrictions on their use than more aggregate data. This is especially true for crime data, which are the focus of this paper. In terms of financial cost, surveys of numerous small areas require more respondents overall than an equivalent survey of fewer larger areas. Secondly, small spatial units are more likely to exhibit ‘small number’ problems (very few incidents) that make it more difficult to isolate signal from noise and determine statistical significance. This is important because low incident counts can lead to significant volatility and, subsequently, unreliability in analyses. And thirdly, many of the previously used socio-demographic and socio-economic variables used in spatial criminology are not available at the micro-place, but at larger census-based units.

For these reasons, choosing the most appropriate spatial unit is very important. As [5] (p 39) put it: “smaller is generally better”, but spatial units that are *too small* are problematic also. So what is an ‘appropriate’ spatial scale? Here, we define the most appropriate scale as that which is as large as possible without causing the underlying spatial units to become heterogeneous with respect to the phenomena under study. The appropriate scale, therefore, balances the theoretical and methodological benefits of working with small units against the difficulty in obtaining small-scale data and the small number problems that can emerge. Here the focus is on crime patterns, specifically, but the proposed method has applicability to other spatial phenomena.

The aim for this work is to develop a general method that is capable of identifying the most *appropriate* spatial unit for the analysis of spatial patterns, if that choice is not predetermined by theoretical considerations or the research question. We then apply the method to the study of various different categories of property crime in Vanouver, BC, Canada, that have different event counts. The proposed approach adapts the multiple resolution goodness-of-fit procedure originally conceived by [6] and combines it with a measure of spatial (dis)similarity—Andresen’s *S* [7–9]—in order to identify the most appropriate scale of analysis, as defined above, for a particular point pattern (crime events in this case). This can then guide the nature of any subsequent analyses. For example, if there is no gain in information from analyzing data at the address than a pre-defined census unit, then the analysis may be undertaken at the census unit with the benefit of having census data available.

It is important to note that the most ‘appropriate’ spatial scale is only relevant for certain types of analysis, namely those that necessarily consider the ‘neighbourhood’ (or a similar aggregate spatial unit) and have an inherently spatial pattern. For example, it makes little sense to aggregate point data if the aim is to study the impacts of different car park layouts on vehicle crime, or the impact of alternative house designs on residential burglary. In these cases it is the specific features of the individual locations that are paramount, and the broader spatial pattern less so. However, if the aim is to better understand how a phenomenon behaves over space and the environmental context is best defined in terms of a neighbourhood, then the spatial scale is important. This necessarily assumes that the spatial process is discrete and changes at area-level boundaries, which is not necessarily a sound assumption, but one that any aggregate spatial study must make as a consequence of representing a spatial pattern at an aggregate level.

## 2 Background

### 2.1 Crime at place, and the concentration of crime events

A substantial body of recent research has found that crime concentrates at ‘micro places’ and, in general, a small unit of analysis is the most appropriate for many quantitative environmental criminology studies [5, 8, 10–13]. These findings, that have emerged through a number of disparate research efforts, were synthesised in a recent systematic review [13] that found consistent support for this “Law of Crime Concentration at Place”, defined by [11] as follows:

“for a defined measure of crime at a specific microgeographic unit, the concentration of crime will fall within a narrow bandwidth of percentages for a defined cumulative proportion of crime” [11]

These findings align both with established environmental theories—that highlight the local nature of criminal events [14–16]—and with the observations of other spatial phenomena where aggregate analyses have been found to hide important lower-level patterns [1, 17]. Although care must be taken when analysing crime at the smallest spatial units (e.g. addresses) because the sparsity of some crime types relative to the number of observation points can artificially inflate the observed concentration [18], broadly most studies report support for the finding of crime concentrates at micro places, and at higher levels than at larger spatial scales [2, 4].

However, determining the appropriate spatial scale is non-trivial for at least three reasons. Firstly, spatial units could be addresses, street segments, or small grid cells, etc. [13], and although smaller units of analysis are typically better able to identify crime concentration [10], does this hold for all crime types (or the number of events in an area to be more general) and all areas? Secondly, high-resolution data that are required for studies at the level of micro-places, such as individual addresses, are harder to obtain than more aggregate data. Thirdly, the difficulty in acquiring fine scale data is further compounded by possible small number problems that occur when rare crime events are analysed at small spatial scales [5]. One could, therefore, ask the question: at what point does it become unnecessary to obtain finer scale data? In other words, are address-level data required for studies of all crime types/counts in all regions, or might more aggregate data be sufficient? What matters is which spatial resolution is *appropriate* for the analysis of a particular phenomena given the nature of the spatial pattern. Or, as [5] put it: “The question then is how small can you go before [the potential problems outweigh] the benefits of more homogeneous areas?” For some phenomena, large neighbourhoods might be an appropriate unit of analysis, whereas for others it might be necessary to use a much higher resolution geography.

To the best of our knowledge, only one paper has investigated a similar research question. [19] attempt to find the spatial unit at which open source geomasked crime data are sufficiently close to the true underlying point pattern (actual police data) to enable meaningful analysis. They use a similar process to that outlined here, albeit only applied to administrative boundaries. Importantly, they found that smaller units of geography (e.g., postal codes) exhibited significantly different spatial patterns when comparing geomasked to non-geomasked crime data. As such, when using geomasked crime data there is a lower bound to which a researcher can trust that the spatial patterns represent reality. Here we investigate the threshold at which finer scales of spatial resolution do not meaningfully add to the analysis when working with various spatial data aggregations.

### 2.2 Goodness-of-fit for spatial point patterns

The method proposed here requires a metric to quantify the difference between two point patterns using an area-based test. Therefore this section reviews some of the most commonly used approaches and outlines the gap that the new method proposed here has been designed to fill.

One way of comparing two point patterns directly is to calculate statistics that describe their first- or second-order properties and then compare the values of these statistics. A common example are nearest-neighbour statistics that examine the average distances between points. For example, the Clark and Evans *R* statistic [20] is the ratio mean minimum distance in a point pattern to that which would be expected under complete spatial randomness. A significance test has also been developed [21]. Therefore by calculating *R* for two point patterns, the degree of clustering can be compared. An improvement over the *R* statistic is Ripley’s *K* function [22] which, rather than only considering a single minimum distance for each point in a pattern, takes *all* of the neighbours that are within a given distance, *d*, into account. The value of *K* at distance *d* is then calculated as the mean of all counts divided by the overall point density. This provides more information about the point pattern as it estimates the amount of clustering present at different distances. Again, graphs of *K*_*d*_ for two point patterns can be compared to estimate similarity between disparate data sets. In addition, the *K* statistic can be adapted such that rather than examining distances between points of the same type, it can compare distances between points of different types (i.e. comparing two different crime types simultaneously); termed ‘cross-*K*’ [23]. There are a number of related statistics that measure slightly different properties of point patterns; see [24] for detail.

The main drawback with these approaches is that they do not provide any information about the differences in the *locations* of the points. For example, two very different datasets could have similar *R* or *K* values if the degree of clustering was the same but the clusters were located in different places. The use of cross-*K* would mitigate this problem as it compares two datasets directly, but as it is a global statistic it does not provide information about *where* the differences between the two datasets occur in space. As an alternative, rather than analysing the point patterns directly it is possible to first aggregate the data to some neighbourhood to create a table or matrix. Administrative boundaries are typically used as the unit of aggregation, but this is not essential. Aggregation has the advantage that, once the data are in tabular form, a range of well-developed goodness-of-fit statistics can be used and, in addition, the cell differences can be mapped to show how the differences vary across space. Relevant statistics include include:

- residual sum of squares—calculated by simply summing the square of the difference between the number of events in each areal unit;
- R^2^—which represents the percentage agreement between a model and the observed data;
- (Standardised) Root Mean Square Error—which has been found to be comparable across spatial systems and not sensitive to large deviations from the mean [25];
- Andresen’s *S* [7–9]—which is adapted for use here.

The main drawback with the typical application of these metrics is that they are usually applied at a single resolution and provide no information about whether the scale of spatial aggregation is appropriate. To overcome this drawback, [6] outline a multiple-resolution method that was developed to measure the similarity between a model of a spatial process and real data. The method was developed for the field of ecology, which is a discipline that has a history of work into appropriate cell (‘quadrat’) sizes. For example, in the 1950s, [26] experiments with the use of different cell sizes with the aim of identifying an optimal size for use in sampling populations of plant species. The authors find that species density is extremely important in the determination of an appropriate sampling cell size, which echoes some of the results discussed here in Section 5.1. More recently, [27] and [21] also discuss the implications for changing cell sizes during sampling. The method that this work builds on most directly, as developed by [6], has been used to assess the reliability of land use change models (e.g. see [28–31]). It has also been used in the field of environmental criminology to assess the reliability of agent-based models [32, 33] but is otherwise not widely used as few studies report to undertake multi-scale validation [34]. By testing the similarity of real v.s. simulated data at multiple resolutions, it is possible to show how well different models perform across different scales. This paper will build on the method proposed by [6] to develop a technique that can be used to assess how similar two data sets are at a given resolution, and estimate the resolution that is the most appropriate for studies of the phenomena.

## 3 Data and methods

### 3.1 Multi-Scale Error Analysis (MSEA) method

To answer the question of the *appropriate* spatial scale, as defined above, this paper presents a new method that builds on a multiple resolution goodness-of-fit procedure [6] and combines it with Andresen’s S Index [7–9, 35] for measuring similarity. The aim is to identify the spatial resolution at which no further disaggregation of areal units leads to new information regarding the spatial patterns within the data: the appropriate scale of analysis. In other words, at what point is there little or no value added in using smaller spatial areal units of analysis? The analysis source code (in the R language) is available as supporting information (S1 File) and online at https://github.com/nickmalleson/Multi-Scale-Error-Analysis/. The source code and required data are both open source, so the analysis presented here can be repeated in its entirety using the source file.

#### 3.1.1 Overview of the method

In short, the method works by taking two point patterns as input and placing a regular grid over them. It aggregates the points to the grid by counting the number of points that fall within each cell from each point dataset. It then calculates the difference between the two point datasets using Andresen’s *S*—through the use of the output for each cell, the test also identifies if one point dataset is increasing or decreasing. Note that if a cell does not have a sufficiently large expected count (as discussed in Section 3.2) then there are too few points to say, with confidence, whether the two point patterns are similar or dissimilar in that cell. In these cases the cell is removed from the analysis and has no influence on the global similarity measure.

**Fig 1:**
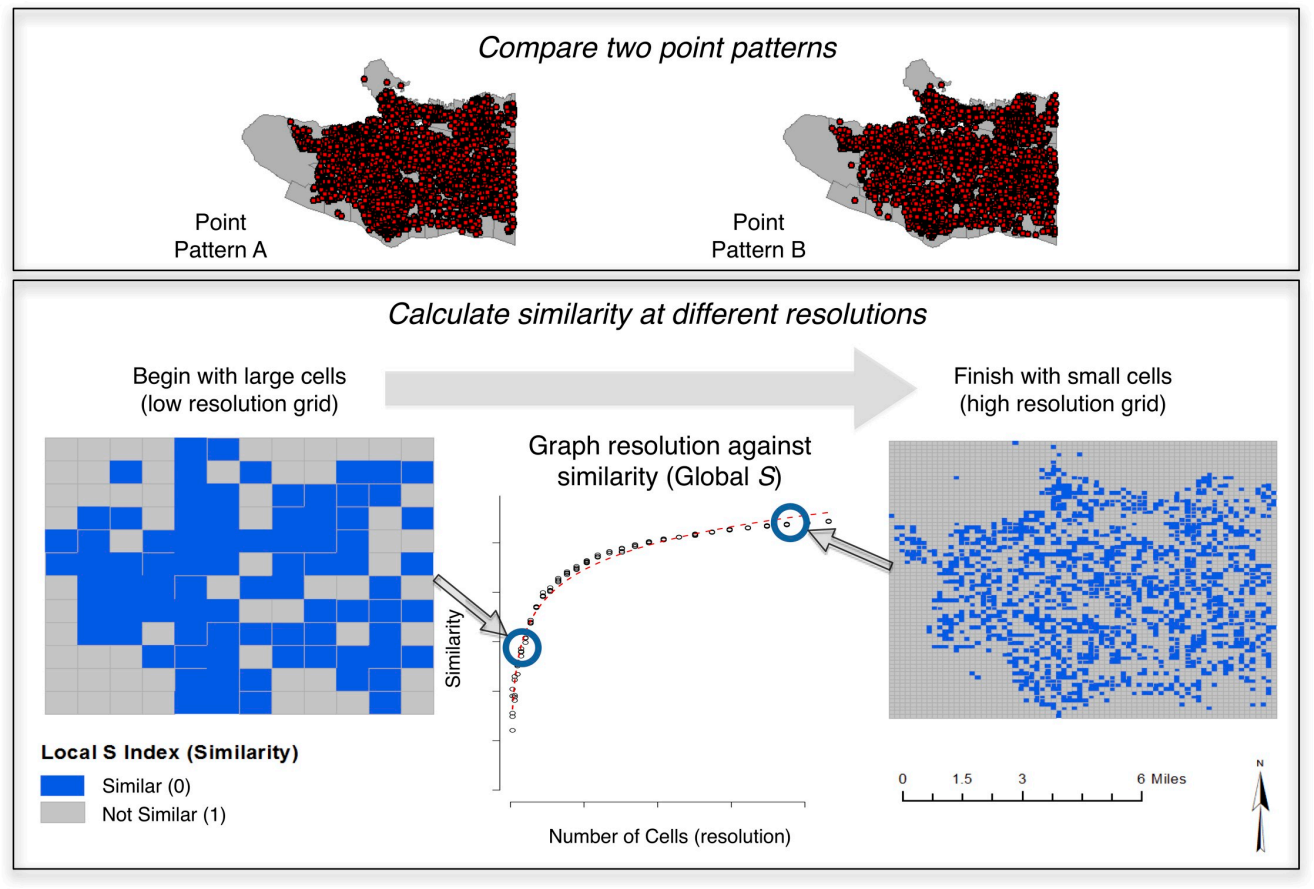
A broad overview of the method proposed here. Grids of varying resolution are placed over two point patterns and the difference between the two patterns can be calculated. The local S Index (*S*_*l*_) (depicted on the maps) indicates the difference between the two point patterns at the cell level, and the Global S (*S*_*g*_) (depicted on the graph) indicates the overall difference at that resolution.

The resolution of the grid is then increased by adding one row and one column of cells, shrinking the grid cells’ size so that the grid covers the point data again, re-aggregating the points to the grid, and then re-calculating the goodness-of-fit. This process is repeated a number of times to allow a comparison of the similarity of the point patterns at the different resolutions in the form of a graph. Fig 1 broadly illustrates the method.

Two point patterns are required for the analysis. The chosen patterns need to be broadly similar (as discussed in Section 3.3) so that differences between them are a result of the choice of the resolution, not an artefact of a difference in the underlying process that produced them. Were there a significant external change (e.g. new social policies, policing interventions, infrastructure developments, etc.) that meant we would expect crime patterns to change, then the two point patterns would not be comparable. Section 3.3 will outline the procedure for choosing comparable crime patterns.

#### 3.1.2 Detailed description

This section outlines the method in more detail. To begin with, it is necessary to decide how many times the resolution of the grid should be increased before the method terminates (termed the number of iterations, *N*). After a certain number of iterations the size of the grid cells will be so small that the difference between the two point patterns (henceforth arbitrarily named ‘*p1*’ and ‘*p2*’ data) will not change as it will be very unlikely for a single cell to contain any points from both data sets. Here we found that *N* = 50 iterations were sufficient, but this will vary by dataset. At each resolution, the grid will also be shifted in a random direction and the points re-aggregated. This reduces the impact of the modifiable areal unit problem [36]. Therefore the total number of grids to create at each resolution, *M*, needs to be decided on. Here we found *M* = 10 to be sufficient; moving the grid more often at each resolution made little difference to the similarity.

The method then works as follows:

1. Draw a bounding box, *A*, that encompasses both point patterns. *A*_*w*_ is its width and *A*_*h*_ is its height. Then calculate the cell width (*c*_*w*_) and cell height (*c*_*h*_) for this iteration such that *c*_*w*_ = *A*_*w*_/*i* and *c*_*h*_ = *A*_*h*_/*i* where *i* is the current iteration number (*i* ∈ {1,2,3,…, *N*}). Note that initially *i* = 1 so *c*_*w*_ = *A*_*w*_ and *c*_*h*_ = *A*_*h*_ (in the first iteration a single cell would span the entire point pattern). As the method iterates, and *i* increases, grids will be made up of smaller and smaller cells.
2. Because the grids will be ‘shifted’ at each resolution (discussed below), it is necessary to slightly increase the size of the bounding box. Otherwise, when the grid is moved, some of the points will be outside the grid and therefore not included in the analysis. To avoid this problem, a slightly larger bounding box, *B*, is created. Its width and height are calculated as: *B*_*w*_ = *c*_*w*_ × (*i* + 1) and *B*_*h*_ = *c*_*h*_ × (*i* + 1) respectively. In other words, the new bounding box will encompass all of the points with enough space for one additional row of cells and one additional column of cells. *B* is then positioned so that it is centred over the points. Note that the increases in the size of the bounding box are not cumulative; in each iteration the box *B* is recalculated by adding some width and height to the first box, *A*.
3. Repeat the following for the number of shifts (*M*) at this resolution:
  - Move *B* in a random direction on the *x* and *y* axes by drawing from uniform random number generators in the range $\left[-\frac{c_{w}}{2},\frac{c_{w}}{2}\right]$ and $\left[-\frac{c_{h}}{2},\frac{c_{h}}{2}\right]$ respectively. This has the effect of shifting the grid randomly but never leaving any points outside of the grid.
  - Create a regular grid *G* by populating *B* with cells.
  - Aggregate the points in the *p1* and *p2* datasets to *G* by counting the number of points in each cell.
  - Calculate the local similarity (*S*_*l*_) between the number of points in both data sets for each cell in *G* using Fisher’s exact test (as will be discussed in Section 3.1.3). Cells that are similar are assigned a value of 1, those that are not similar are assigned a value of 0.
  - Calculate the *expected* count for each cell (see Setion 3.2). This is used to identify cells that have too few points to say anything statistically significant about the similarity between the two data sets.
  - Calculate the global similarity between the two datasets for this iteration (*i*) and shift (*j*). The Global *S* Index (*S*_*g*_) is used here and is calculated as the mean of the local similarity values:
    $$\begin{array}{c} S_{g}=\frac{\sum_{k=0}^{C}S_{l}\left(k\right)}{C} \end{array}$$ (1)
    where *C* is the number of cells and *S*_*l*_(*k*) is the local S index for cell *k*. As *S*_*l*_(*k*) ∈ [0, 1] then 0 ≤ *S*_*g*_ ≤ 1 where larger values (*S*_*g*_ → 1) indicate greater similarity. Note that cells with an insufficient count (as per step 3e) are not considered when calculating *S*_*g*_, so for grids with small cells it is likely that *C* will be smaller than the total number of cells in the grid.
4. Repeat from (1) until the maximum number of iterations has been reached (*i* = *N*), or until the change in similarity from one iteration to the next becomes negligible. Both of these cutoffs will depend on the specific application.
5. Plot the *S* index against the number of cells at each resolution and estimate the point at which the change in error is sufficiently small as to indicate the appropriate resolution has been reached.

The output of the method is a total of *N* × *M* regular grids (where *N* is the total number of iterations and *M* is the number of shifts at each iteration) showing similarity at varying resolutions.

#### 3.1.3 Measuring global similarity: The global *S* index (*S*_*g*_)

Once the point patterns have been aggregated to a particular spatial grid (step (3f) above), it must be decided how to quantify the overall difference between the two grids. In their original paper, [6] develop their own measure of error that is appropriate when categorical spatial data (e.g. land use categories), as are commonly used in ecology, need to be aggregated. Here the number of points in an area are counted to aggregate them, ultimately creating ratio data, so therefore any suitable measure of similarity can applied. These include the standard coefficient of correlation (R^2^), the (standardised) root mean square error, or many others as discussed in Section 2.2.

Here we use the global *S* index (*S*_*g*_). The *S*_*g*_ index is a cell-by-cell comparison of point patterns *p*1 and *p*2. The test has traditionally been performed using a Monte Carlo permutation test (e.g. [7, 8, 37]), but more recent work suggests that the use of a Fisher’s exact test is more appropriate [9], which is the approach taken here. *S*_*g*_ is used over others for a number of reasons. Firstly, the test is non-parametric, where as others like *R*^2^ or the RMSE are parametric, and hence make assumptions about the underlying data distributions which are not necessarily appropriate here. Secondly, *S*_*g*_ is derived directly from the local version of the test (*S*_*l*_), which provides an estimate of similarity of *individual cells*. The need to distinguish local variation across grid cells rules out the use of alternative non-parametric tests, such as Kendall or Spearman, which are global measures. A step-by-step description of the method is available in the supporting information (S1 Appendix).

### 3.2 Statistical significance and quantifying local ‘similarity’

As spatial units decrease in size, there will be fewer points per cell than compared to larger units. It is not possible to determine whether the number of events that occur in a cell from two point patterns are significantly different if the numbers of events are too small. What should be done with grid cells with such low counts such that they cannot be distinguished with any statistical significance? If these grid cells are simply marked ‘similar’ they will inflate the global similarity artificially, resulting in perfect similarity when using very small grid cells. For this reason these grid cells are removed from the analysis completely, thus focussing on the cells for which meaningful differences can be found. To make the decision on which cells to remove, the standard recommendation for the Chi-squared test is used as follows.

Consider the case where there are *n* spatial units, *U*_1_, *U*_2_,…, *U*_*n*_ and two point patterns: *X*_1_ and *X*_2_. It is then possible to estimate the *expected* number of points from *X*_1_ or *X*_2_ in any given unit, *U*_*i*_, by multiplying the row and column marginals and dividing by the grand total. For example, to calculate the expected number of events from *X*_1_ in unit *U*_*i*_:

$$E_{X_{1},U_{i}}=\frac{\overset{\text{Row}\;\text{marginal}}{\overset{︷}{X_{1}\left(i\right)+X_{2}\left(i\right)}}\times \overset{\text{Column}\;\text{marginal}}{\overset{︷}{\sum_{i=0}^{n}X_{1}\left(i\right)}}}{\underset{\text{Grand}\;\text{total}}{\underset{︸}{\sum_{i=0}^{n}X_{1}\left(i\right)+X_{2}\left(i\right)}}}$$ (2)

where *X*(*i*) denotes the number of points from process *X* in unit *i*. When the number of units, *n*, is large, it will be the case that for many units *U*_1_, *U*_2_,…, *U*_*n*_ the number of points per unit will be very low, *U*_*i*_ ≪ *n*. Following standard recommendations the threshold used here is set a 5, such that any cell with less than an expected frequency of 5 (either $E_{X_{1},U_{i}}$ or $E_{X_{2},U_{i}}$) is excluded. This is a conservative test [38], so the cut-off of 5 could be relaxed in future work.

### 3.3 Data

The data used here represent calls or complaints made to the Vancouver Police Department. They are publicly available through the City of Vancouver Open Data Catalogue (https://data.vancouver.ca/datacatalogue/crime-data.htm) and are available from 2003 to the present under various crime categories. The data are anonymised prior to being made availability on the portal. Here, the following four crime categories have been chosen:

1. Breaking and entering—residential (BNER);
2. Breaking and entering—commercial (BNEC);
3. Theft from vehicle (TFV);
4. Theft of bike (TOB).

These four types have been chosen because: they are voluminous (each has at least 2000 events per year) so there is value in applying quantitative methods; they represent crimes that are likely to be reported due to the need to make insurance claims so should be largely representative of the true crime distribution; they have very different spatial patterns (some are much more heavily clustered than others) so represent a good test of the method.

For each crime type, two data sets are required for a comparison. Here, data from 2015 will be compared to those for 2016. It was decided to use 12 months worth of data to ensure that there are a sufficiently large number of points for meaningful quantitative analysis and because 12 months is a time period that is commonly chosen in the field to mediate the impacts of seasonality on crime patterns. It is important that during the comparison period (Jan 2015–Dec 2016) the crime patterns do not change radically, otherwise the method will identify these changes rather than the inherent randomness that will always be present in the study of social phenomena. During the time period chosen there were no major policy or policing interventions that would suggest the patterns should be radically different. Although it is beyond the scope of this paper to attempt to separate the influence of the random fluctuations in the pattern from any longer-term systemic changes, future work will attempt to derive a more precise definition of the point at which wider systemic changes obscure the natural fluctuations (i.e. more precisely defining the point that they become ‘radically different’).

**Fig 2:**
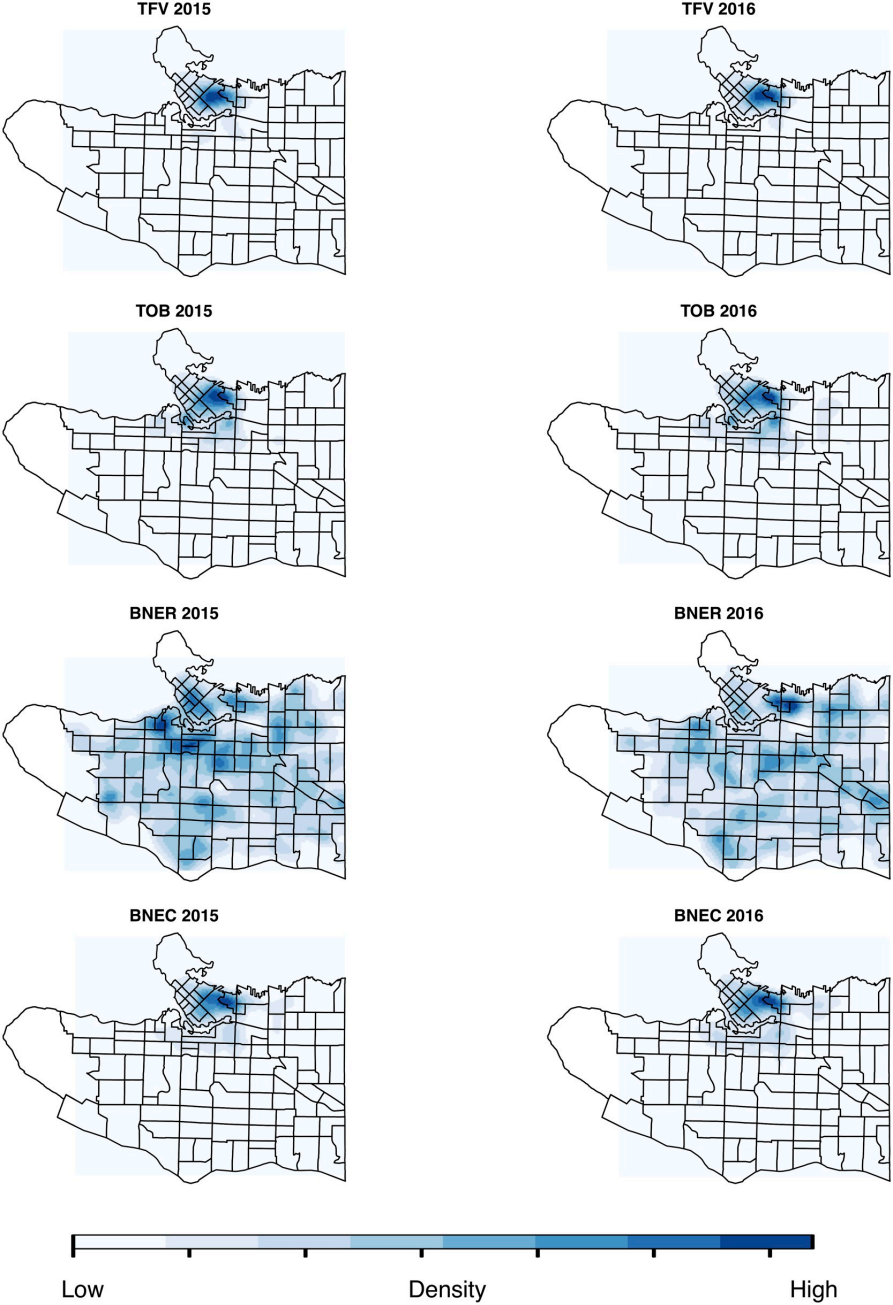
Densities of the four different crime types in the two years (2015 and 2016) used in the analysis. Census tract boundaries have been overlaid for context, although these are not used in the analysis.

Fig 2 illustrates the density of each of the crime types in 2015 and 2016. Although most are similar (as required), residential burglary (BNER) does appear to have undergone some changes in the two years. It is also much more widely distributed throughout the study area than the other crime types. Both of these factors have interesting implications for the results, as Section 5 will discuss.

To assist with interpreting the size of the cells in the different grids, six different grid resolutions are overlaid on to some of the results graphs in the later sections. The resolutions of these grids range from approximately 6Ha (slightly smaller than a dissemination area; the smallest Canadian census unit) to 100Ha (roughly equivalent to a census tract). These resolutions do not influence the analysis itself, but just illustrate how the size of the cells in different grids relate to the size of city blocks.

## 4 Results

### 4.1 A simulation of point similarity

In order to better understand how the method will behave when applied to real spatial data, it is first applied to some simulated point patterns. Five different point patterns are created using an inhomogeneous Poisson process as implemented using the spatstat::rpoispp R function. The formula used to determine the density of the points per unit area is:

$$\begin{array}{c} a*exp(-b*x)*exp(-c*y) \end{array}$$ (3)

where *a* is a balancing factor used to determine the approximate number of points created (larger values for *a* result in more points), and *b* and *c* determine the distribution of the points in the *x* and *y* planes. Larger *b* and *c* values mean that the points are more clustered to the lower end of the scale. For example, the first point pattern created (*P*1) has *b* = *c* = 1 so points are weakly clustered in the lower left corner. As *b* and *c* increase the points become more clustered. The clustering itself is not important, what matters is the difference between the patterns; this can be controlled by varying *b* and *c*. *a* is chosen so that each pattern has approximately 3,000 points, which is a similar number of events to the crime data. Table 1 describes the point patterns that were created to run the simulations. *P*1 and *P*2 are the most similar as they are created using identical parameters. *P*3, *P*4, and *P*5 become increasingly dissimilar.

**Table 1:** A description of the simulated point patterns used to test the algorithm. The parameter *a* is chosen so that each point pattern contains approximately 3,000 points. Parameters *b* and *c* determine the amount of clustering; larger numbers produce more clustering.

Name	Explanation	Parameter values (*a*, *b* and *c*)
*P*1	The ‘base’ data set. All others are compared to this one	*a* = 8000, *b* = 1, *c* = 1
*P*2	Created using an identical formula to *P*1 (the most similar)	*a* = 8000, *b* = 1, *c* = 1
*P*3	Patterns become	*a* = 12000, *b* = 1.5, *c* = 1.5
*P*4	increasingly different	*a* = 17000, *b* = 2, *c* = 2
*P*5	to *P*1	*a* = 32000, *b* = 3, *c* = 3

**Fig 3:**
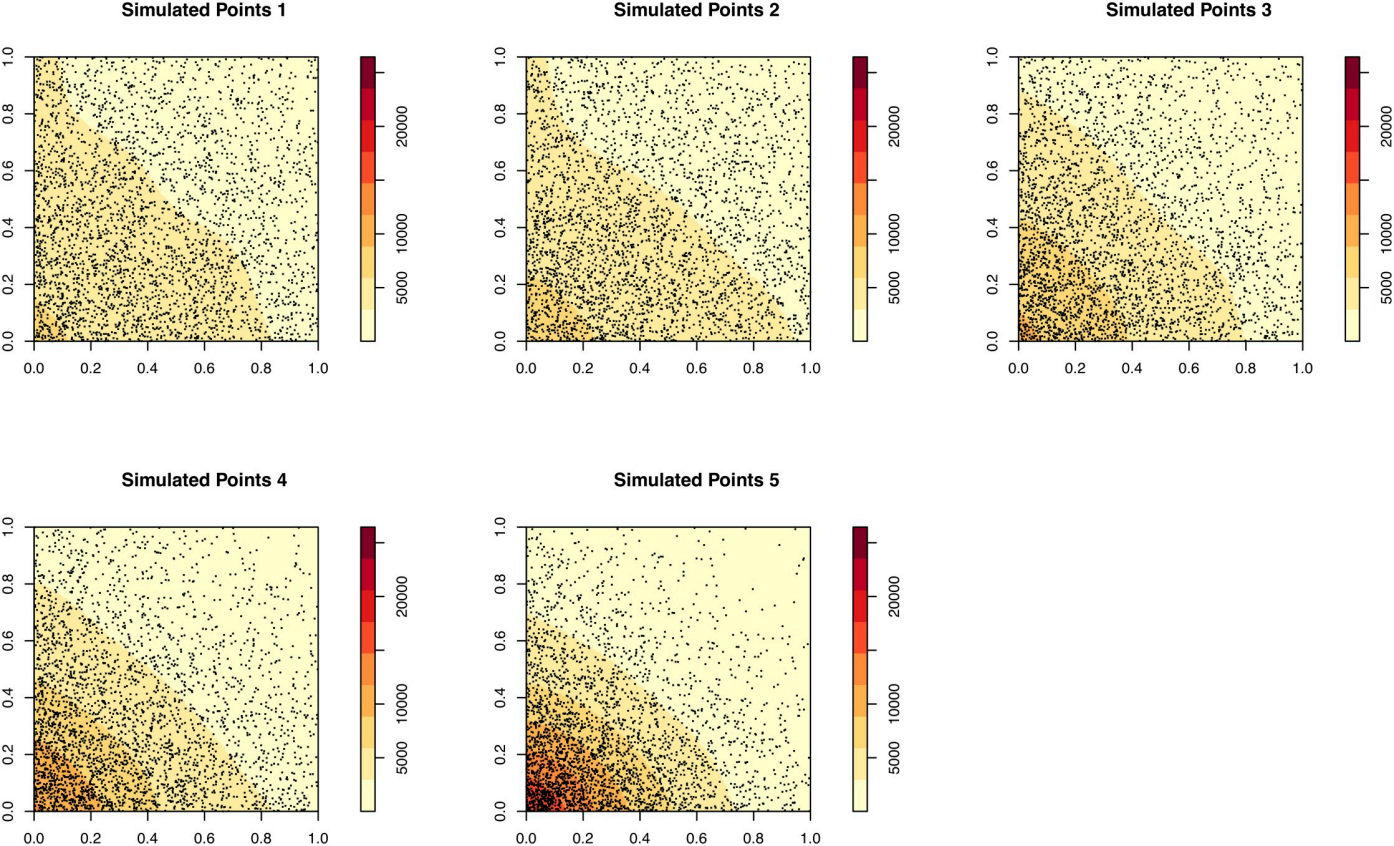
The five simulated point patterns used to test the algorithm. *P*1 and *P*2 are the most similar (they were created using identical parameter values). The remainder (*P*3, *P*4, *P*5) become increasingly dissimilar.

**Fig 4:**
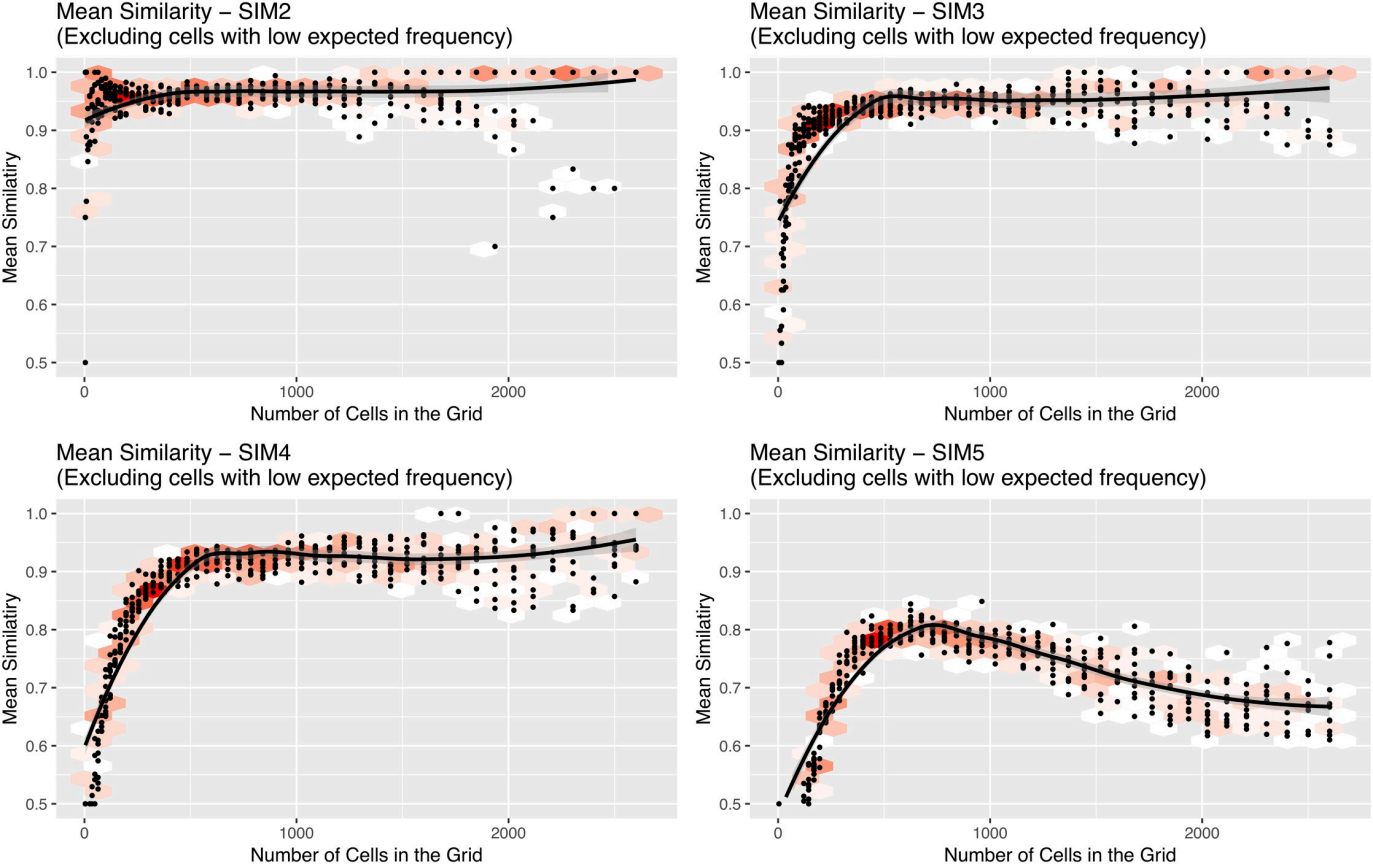
A scatter plot of the global similarity (*S*_*g*_) of all cells (i.e. the mean of all individual cells’ similarity) at each resolution. All comparisons are made to *P*1 (SIM2 compares *P*1 to *P*2, SIM3 compares *P*1 to *P*3, and so on). Points in the scatter plot have been aggregated into hexagonal bins to aid interpretation; red hexagons contain the largest numbers of scatter points. Lines of best fit and 99% confidence intervals are estimated using Local Polynomial Regression Fitting [39] (as implemented in the stats::loess R package).

Four experiments are conducted in total. In each experiment, *P*1 is compared to one of the other simulated patterns. Fig 3 illustrates the density of each of the point patterns and Fig 4 illustrates the variation in similarity.

With all point patterns, the mean similarity initially increases. This is an unintuitive result as similarity could be expected to decrease with cell size because it becomes less likely to find points from both datasets in the same cells. The increase occurs because with very large cells, some (i.e. those near the origin) will have a very large number of points in them when compared to cells that are located nearer the outskirts of the simulation boundary. We observe an increase in the proportional difference with increasing number of points, such that if there are cells with very large numbers of points, they will have a large statistically significant proportional difference. This reduces the similarity for large grids. After reaching a peak, the mean similarity then remains relatively consistent or begins to decrease. Importantly, it is immediately obvious which point patterns are the most similar. In simulation 5 (which compares the least similar point patterns—*P*1 and *P*5) the similarity takes considerably longer to reach its peak, does not reach such a high level, and declines afterwards.

### 4.2 Expected crime frequency

**Fig 5:**
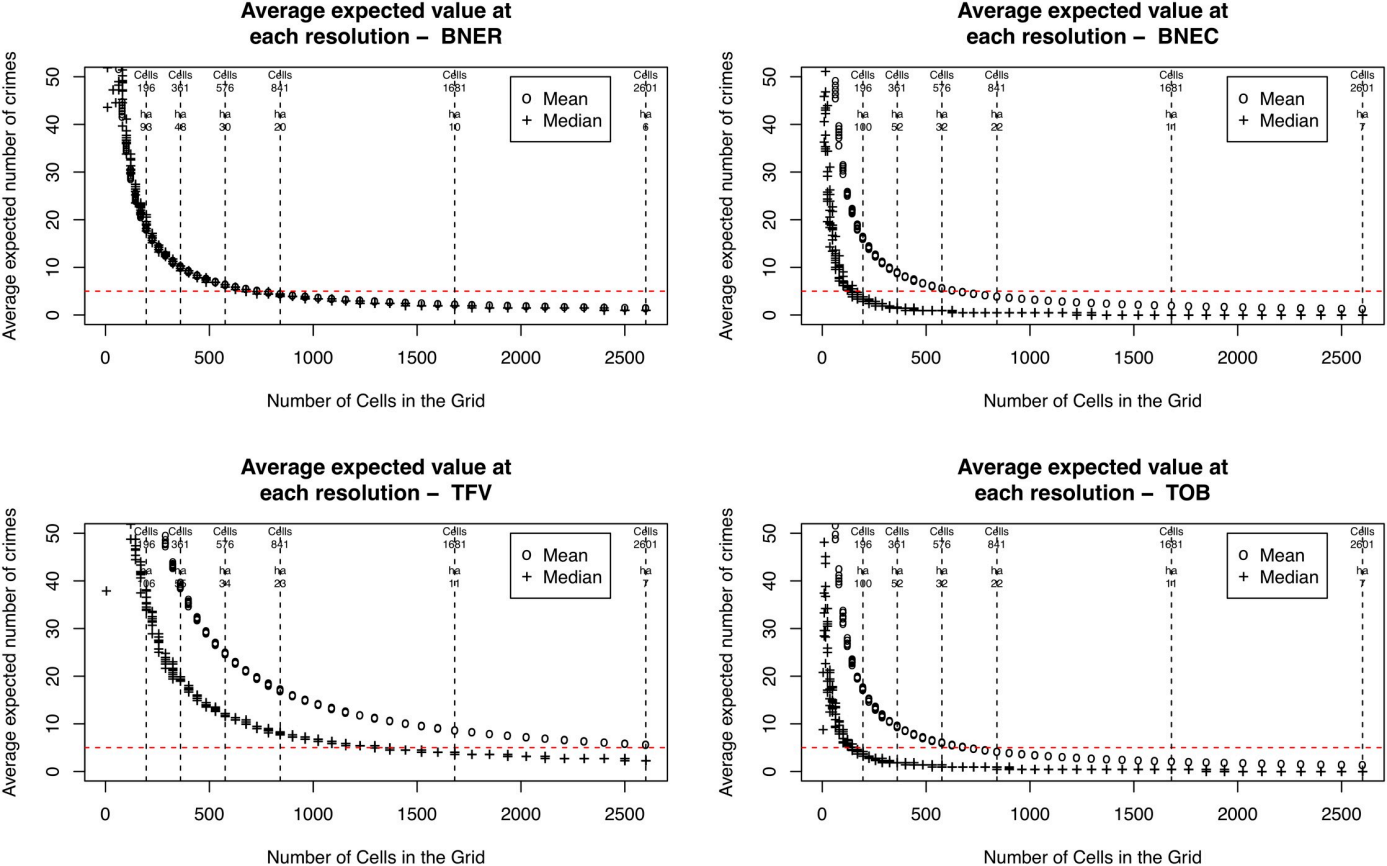
Graphs illustrating the change in average expected value at each resolution for the four different crime types. Note that the horizontal line illustrates the cut off at which point individual cells would not have a sufficiently high expected value to draw statistically significant conclusions. The vertical lines denote some different resolutions used for illustrative purposes.

Before reporting the results of the method applied to the real crime data, it is useful to better understand how the spatial scale (i.e. grid resolution) affects explanatory power. Fig 5 illustrates the mean expected value at each resolution. This is constructed by first calculating the expected value (discussed in Section 3.2) for each cell, and then calculating the mean expected value across all cells at that resolution. Assuming that crimes are reasonably uniformly distributed across the study area, then grids with a higher mean value have a greater number of cells with a sufficient number of points to be able to calculate similarity. Those with lower means will have fewer cells with sufficient explanatory power. Whilst not directly relevant to the main results of the paper, the *distribution* of expected values could, potentially, reveal interesting information about the underlying spatial distribution of the crime patterns. For example, where a crime pattern exhibits a larger mean than median, this implies that the points are clustered; the mean is skewed by a few cells with very large numbers of points in them.

**Fig 6:**
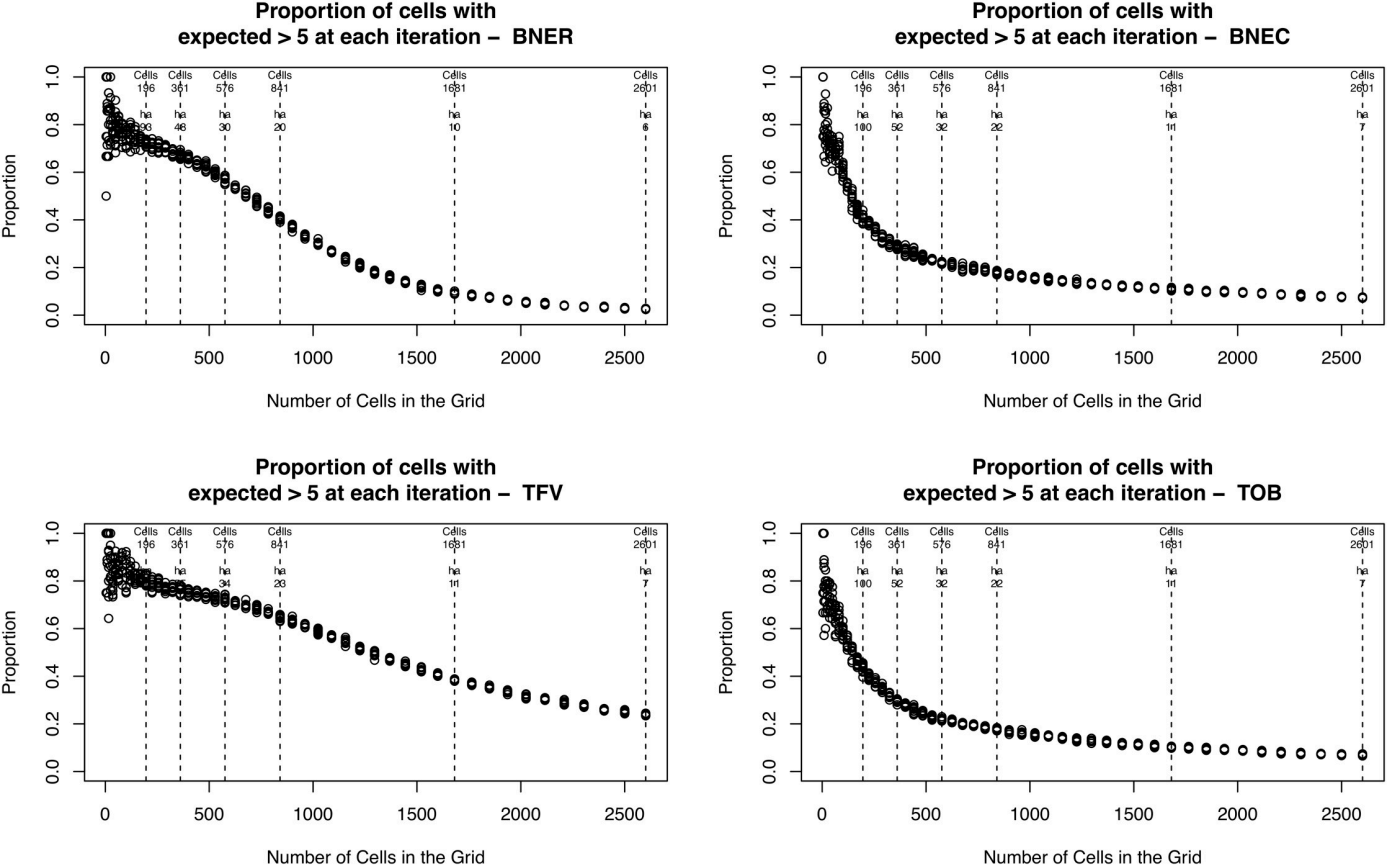
The proportion of cells at each resolution that have a sufficiently large number of expected crimes in order to assess their similarity. The vertical lines denote some different resolutions used for illustrative purposes.

Fig 6 provides more information about the expected frequency by illustrating the *proportion* of cells at each resolution that have a sufficiently large expected value in order to be able to estimate the similarity between the two point patterns. This is useful because it allows for statements such as “at resolution *a*, *b* percentage of all cells are used to estimate the overall similarity”. Where *b* starts to become very low then it becomes difficult to make statements with confidence about overall similarity because there will be few cells actually used in the calculation. Again, this is not directly relevant to the results here, but could be useful as a means of estimating the spatial unit of analysis at which there will be, on average, an insufficient number of events to test properly for local spatial differences *a priori*.

### 4.3 Overall similarity changes

**Fig 7:**
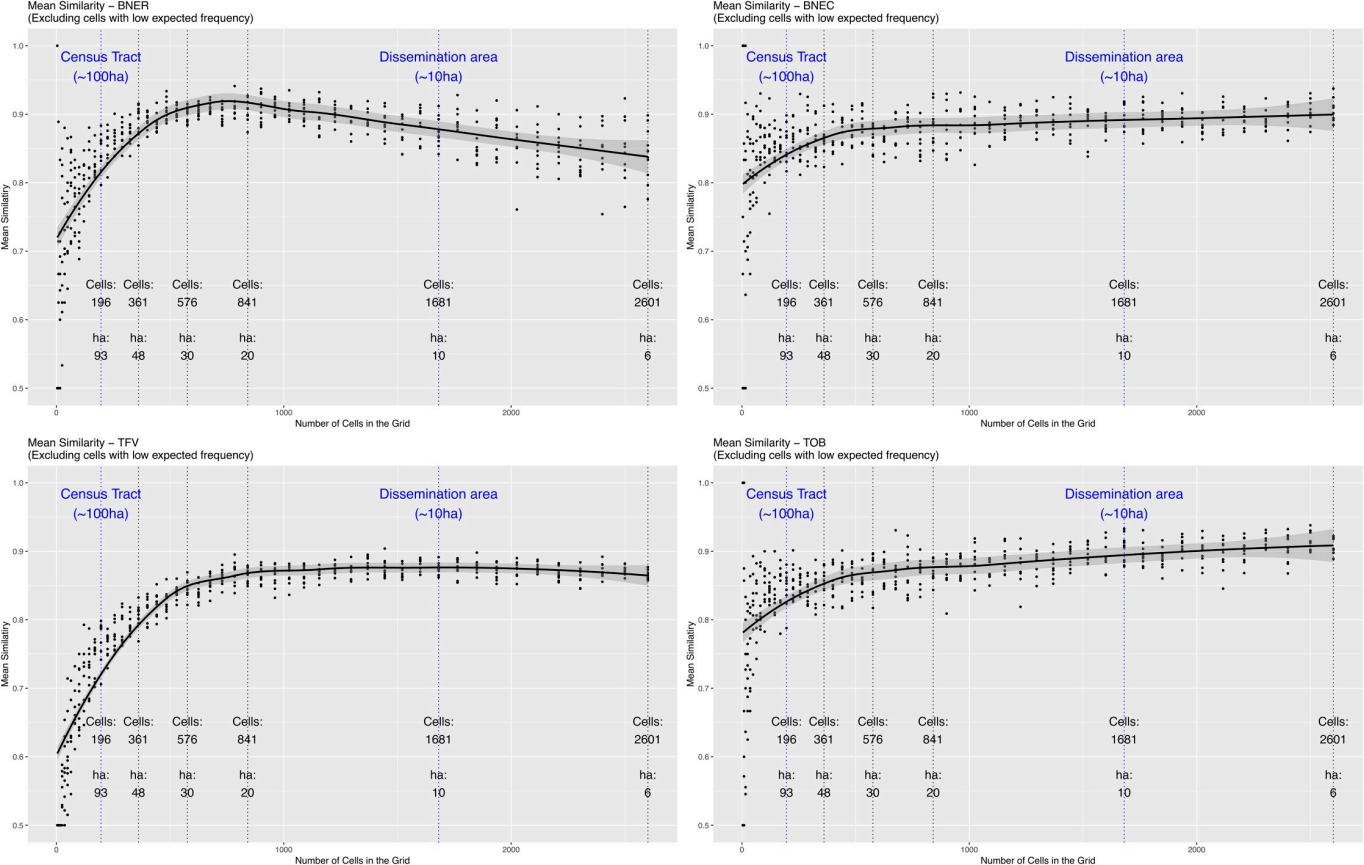
A scatter plot of the global similarity (*S*_*g*_) of all cells (i.e. the mean of all individual cells’ similarity) at each resolution. Lines of best fit and 99% confidence intervals are estimated using Local Polynomial Regression Fitting [39] (as implemented in the stats::loess R package). The vertical lines denote some different resolutions used for illustrative purposes.

Having outlined the proportion of cells that, at each resolution, have a sufficiently high number of points to make a comparison between the two crime patterns, Fig 7 illustrates how the mean similarity changes with resolution. With all crime types, the mean similarity initially increases in a similar manner to that of the simulations. After reaching a peak, the mean similarity then remains relatively consistent or (as in the case of residential burglary) decreases. These results are discussed in detail in Section 5.1.

## 5 Discussion

The aim for this work is to develop a general method that is capable of identifying the most appropriate scale for the analysis of spatial patterns, defined as that which is as large as possible without causing the underlying spatial units to become heterogeneous with respect to the phenomena under study. The method proposed here does this by creating iteratively smaller areas and, at each scale (or ‘resolution’), determining the similarity between two point patterns that should be spatially similar.

### 5.1 The most appropriate spatial scale

**Fig 8:**
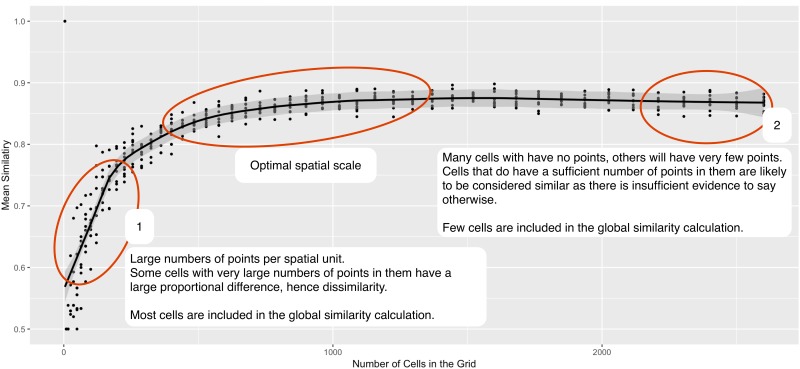
An explanation of the change in similarity with the size of the spatial unit.

Identifying the most appropriate scale is non trivial. As the size of the spatial units changes, there are two distinct statistical processes that take place. These, as illustrated in Fig 8, are:

1. Initially, the point pattern similarity is low, and it increases as the number of cells increases. This is because when there are very large spatial units, some cells have very large numbers of points in them. We observe an increase in the proportional difference with increasing number of points, such that these cells with large numbers of points (compared to others on the outskirts of the study area that have fewer) have a statistically significant proportional difference that is not necessarily large in magnitude. This reduces the similarity for large grids.
2. At the other end of the scale, where there are many small cells, there will be few points in each cell. This has the effect of widening the confidence intervals and subsequently making it less likely that there is a significant difference. This *artificially amplifies* the similarity. To remedy this we remove individual cells that have insufficient explanatory power (as per Section 3.2), otherwise a point pattern would converge on total similarity (*S*_*g*_ → 1). The removal of these cells means that although the similarity does not tend to 1, at the highest resolution grids only a small proportion of cells contribute information to the global similarity measure.

The end result of these two processes are the graphs that were illustrated in Fig 7. The most appropriate spatial scale can be found somewhere between the two extremes of the graph. The pertinent question therefore becomes: *where, in the region between the two extremes, does the most appropriate spatial scale lie?* Interestingly, the behaviour of the global similarity varies by the crime type in question, and therefore the explanation needs to be tailored for each one.

- **Residential Burglary (BNER)**. With BNER, there is a peak in similarity when cells are approximately 20 hectares in size. This size, which is close to twice the square area of a dissemination area (the smallest Canadian census neighbourhood) appears to be the most appropriate spatial scale at which to analyse the phenomenon. Of course, using larger units will hide lower-level patterns so generally smaller is better (the contemporary ‘crime at place’ literature is clear on this). But, importantly, these results suggest that when using units that are much smaller than 20 hectares the *noise*, that will always be present with such spatial phenomena, is captured instead of the *signal*. In other words, there are so few points in cells below this size that we can expect significant volatility and, subsequently, unreliability in analyses.
  It is, however, important to recall that the 2015 and 2016 BNER point patterns are not as similar as those of the other crime types. Therefore some of the dissimilarity is caused by wider spatial variations across the two years (i.e. crime hotspots moving) rather than purely due to the underlying randomness in residential burglary. Were the patterns more similar then the most appropriate spatial scale would be smaller. Future work can investigate this particular case in more detail, particularly with respect to better understanding why the spatial distribution across the two years is not more consistent.
- **Theft from Vehicle (TFV)**. In this case, the similarity increases until the spatial units are approximately 20ha and then plateaus. With TFV, therefore, there appears to be no *disadvantage*, in terms of signal v.s. noise, to using spatial units that are smaller than 20ha. However, it is worth noting that as the cell size decreases there will be fewer cells with sufficient explanatory power to contribute to the global similarity. For example, from Fig 6, approximately 65% of all cells have sufficient power when the size of the spatial units is 20ha, but this decreases to around 40% with cells of 10ha (roughly equivalent to a dissemination area).
- **Commercial Burglary (BNEC)** and **Theft of Bike (TOB)**. As with BNER and TFV, the similarity increases rapidly initially but then, rather than plateauing, it continues to increase gradually. This suggests that, even with cells of only 6ha, there is still a signal that can be identified above the background noise. However, as with the other crime types, the number of cells with a sufficiently large number of crimes will be very low at these fine resolutions. This result is most likely to be a consequence of the degree of clustering that these two crime types exhibit; a point that will be elaborated on further shortly.

A further important consideration is the number of points in a dataset and, similarly, the point density. As the number of points increases then, if everything else remains equal, the appropriate spatial scale becomes finer. This is because with two *similar* patterns, more points means that the cell size will be able to become quite small before noise begins to hide the signal. This is important because phenomena that occur frequently, or at least frequently in a specific area (e.g. a highly clustered phenomena) can be reliably disaggregated to quite small cells, at least in the areas where the phenomena occurs densely. The ‘appropriate spatial scale’ not only depends on the abundance of the phenomena itself, but also the degree to which it is clustered.

### 5.2 Spatial distribution of similarity

Section 5.1 discussed the potentially *most appropriate* spatial scale for each crime type. But what does this look like in practice? To provide some context, this section explores the spatial distribution of the local similarity measures at a few different scales. The focus is on residential burglary (BNER) and theft of bike (TOB) exclusively, as these two crime patterns have very different spatial structures and, hence, demonstrated very different similarity profiles (as per Fig 7).

To begin with, it is important to consider that that BNER is much more widely distributed throughout the study area than TOB (as was evident in Fig 2). This is entirely expected given the contemporary environmental criminology explanations for observed crime patterns and their relationship with the distribution of potential targets. In short, residential properties are widely distributed, but bicycles are much more likely to be available (and accessible) outside transport hubs or places of employment. The interesting thing to note about these different degrees of clustering is the impact that they have on the most appropriate spatial resolution.

**Fig 9:**
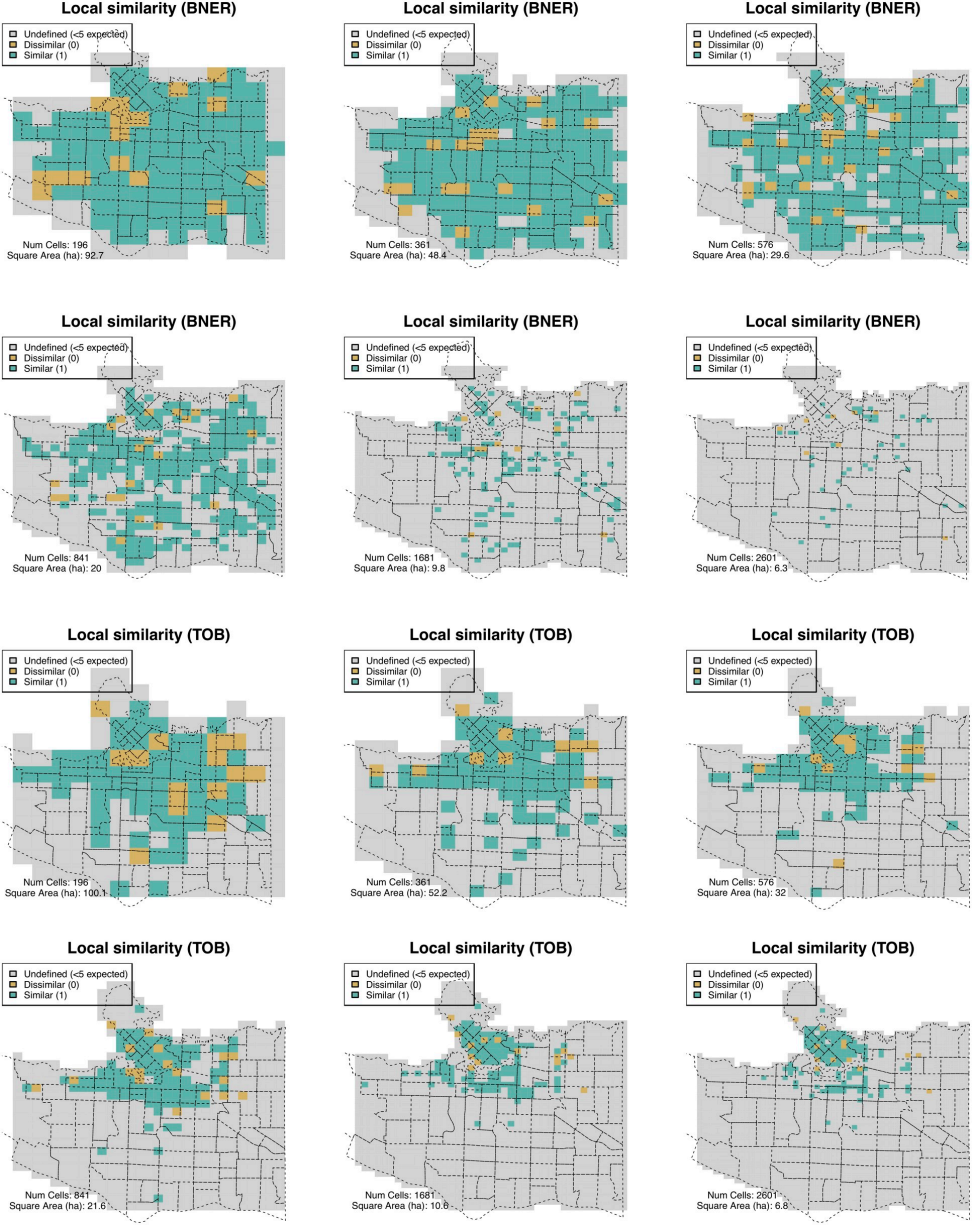
The similarity of residential burglary (BNER) and theft of bike (TOB) in 2015 and 2016, after ignoring cells with insufficient information to make a statistically significant comparison.

Fig 9 illustrates six grids at the illustrative spatial scales that were chosen in Section 3.3. It shows the local similarity values for BNER and TOB at the different resolutions, where 1 implies similarity in the cell and 0 implies no discernible difference. Cells with insufficient explanatory power are also highlighted.

It is immediately apparent that, as outlined earlier, the number of cells with sufficient explanatory power for burglary drops rapidly once cells become smaller than 20ha. With cells of 10ha, there are very few cells that have a sufficiently large number of points to determine, with confidence, whether the two point patterns are similar or dissimilar.

Regarding TOB, although the majority of the study area does not exhibit sufficiently large expected values, there is a consistent spatial pattern in (dis)similarity in the central business district and surrounding transport hubs, even down to cells that are under 10ha (less than a single dissemination area). In these areas, the appropriate spatial scale is very small indeed. These results therefore suggest that there is value in obtaining extremely high resolution data because even with very small spatial units there is still sufficient signal above the background noise to warrant empirical analysis.

### 5.3 Is ‘smaller’ really ‘better’?

On the whole, recent research in environmental criminology has pointed towards the importance of ‘micro places’ in crime analysis [5, 10–13]. It is important to explain here that these findings do not reject that assertion. High quality, disaggregate data will always be preferable to more aggregate data. At the very least, disaggregate data can always be aggregated. For example, current work that examines the impacts of the road network [40, 41] on crime occurrence would most likely obfuscate important micro-level features were the data aggregated to neighbourhoods. This situation holds in other fields.

This work does not recommend aggregating point data to the ‘most appropriate’ spatial unit. Rather, the purpose of this work is to provide a framework that can be used to, among other things, explore the scale at which the *broader spatial patterns* start to become overshadowed by random noise. The is only relevant for research that is specifically interested in spatial patterns, not for similar research that, although spatial in nature, does not explicitly consider *space*. In the circumstances where the broader spatial patterns are of interest, the method presented here can be useful in two respects. Firstly, in the cases where data that have been aggregated to a spatial neighbourhood are available, this method can be used to estimate whether there would be value in attempting to obtain finer-scale data. The method could be performed by the data controllers to look for evidence for the need to release higher resolution data to the analyst. Secondly, for studies that do require aggregate data (such as those that explore neighbourhood-level phenomena) it can provide evidence for the most appropriate spatial unit to use in the aggregation—i.e. not so large that the scale causes lower-level patterns to be smoothed out, but not so small that there are insufficient events per unit to make any statistically significant assertions.

### 5.4 Meaningful spatial units

Before concluding the paper, it is worth briefly discussing the implications of using rectangular grid cells as the underlying spatial unit. A body of work in environmental criminology, as well as in other geographical fields, has attempted to identify the most *meaningful* spatial unit for the analysis of spatial phenomena. For work on social phenomena, these units are typically defined in a way that associates them explicitly with the underlying space, i.e. one that connects with how the infrastructure shapes people’s travel patterns, communication, sense of community, etc. For example, recent work on street segments has been particularly illuminating [40–44]. A regular grid of cells, such as that used here, does not meaningfully link to the underlying phenomena. This is a drawback because it is well known that the *real* underling geography will have significant effects on the level of crime. Therefore future work could design an algorithm to create more *meaningful* units at various different resolutions—using (for example) physical boundaries, social data, and land-use information—in a similar manner to those that are used to create census and other administrative boundaries currently. Such an effort is well beyond the scope of this work though, and it is worth pointing out that similarly meaningless spatial units, such as postcodes, are also used regularly and do not reflect real ‘neighbourhoods’. Finally it is also worth mentioning that the use of grids will also probably lead to ‘zoning effects’ [5] because the zones have not been drawn with the deliberate intention of maintaining homogeneity as is typical with the creation of administrative boundaries. Again, future work that explored more nuanced mechanisms for creating appropriate / ‘meaningful’ spatial units should account for zoning effects as well.

## 6 Conclusions and future work

This paper has presented a new approach that can be used to identify the most *appropriate scale* at which to aggregate point data to areas. It does this by creating a number of regular grids with different resolutions (in a similar method to that of [6]) and estimating the *similarity* between two realisations of the point pattern at each resolution. The most ‘appropriate’ scale is the one that balances the benefits of using smaller spatial units (larger units will probably hide important heterogeneity that is apparent at higher resolutions) against the drawbacks (including difficulties in obtaining fine spatial data in the first place, as well as the risks of the underlying pattern being obscured by noise). The method is applied to the study of crime patterns in Vancouver, Canada, and the results provide evidence for the size of spatial unit that is the most appropriate for the different types of crime studied (these were residential burglary, commercial burglary, theft from vehicle and theft of bike). The method is dependent on both the size of the point patterns (the number of events) and the degree of clustering, so it is doubtful that a single ‘appropriate’ scale will ever be identified for a phenomenon. But the method is nevertheless useful as a means of better estimating what spatial scale might be appropriate for a particular piece of analysis.

There are some interesting future directions that could be taken. In their original paper, [6] discuss the possibility of using their method to compare time series data. They do this by aggregating the fit across all resolutions to create an overall measure of similarity for two given data sets in a given time period, and repeat this process for data that span a number of years. There is obvious potential to extend the method proposed here to spatio-temporal data and examine how crime patterns (or other dynamic spatial phenomena) have changed over time. This could be coupled with an extension to the *S* index that takes temporal similarity into account as well [45, 46]. Similarly there are further experiments that could be conducted to better understand how the number of events in a point pattern, including different methods to subset the point pattern, and/or the density of these events, affects the similarity measure.

One of the main caveats with the work is that the spatial units used—regular grid cells—are not *meaningful* with respect to the underlying phenomena (cities are not organised into regular cells). Therefore future work could begin to experiment with spatial units of different shapes. Simple extensions could be to use more appropriate regular shapes like hexagons, and then a more nuanced method could be developed that took account of the underlying urban backcloth (e.g. road networks, community boundaries, etc.). Additionally, in the current context, crime data may not be analysed in isolation. If crime are being analysed with another point data set, the locations of business as risk factors, for example, this latter variable variable must also have its appropriate spatial scale identified to see if it corresponds to the appropriate scale for the crime data. As such, future research should consider this research question in a bivariate context as well.

## Supporting information

S1 File Source code (R markdown). The empirical work outlined here can be conducted, in full, using the R source code included in the publication and also available at: https://github.com/nickmalleson/Multi-Scale-Error-Analysis. It is in ‘R Markdown’ format so can either be used to generate an html file with text and graphics included, or the R commands can be run independently. The script depends heavily on the *sppt* R package [35]. The script attempts to download the required crime data (that are open source), so all experiments outlined here can be run in their entirety. The only data that are required are Vancouver administrative boundaries that are used for drawing maps. They are available here: https://github.com/nickmalleson/Multi-Scale-Error-Analysis/tree/master/vancouver_boundaries. The files must be downloaded and placed in a directory called vancouver_boundaries in the same location as the script. The easiest way to run the script is to load it into RStudio and run it from there. Alternatively, it can be run from a bash command line to generate an html file using this command (all on a single line):
Rscript -e “require(knitr); require(markdown);
knit(‘multi_scale_error_analysis.Rmd’,
‘multi_scale_error_analysis.md’);
markdownToHTML(‘multi_scale_error_analysis.md’,
‘multi_scale_error_analysis.html’);”
(ZIP)

S1 Appendix The method to calculate Andresen’s *S*. (PDF)
