# Supplementary material for: Identifying the appropriate spatial resolution for the analysis of crime patterns
Source: PLoS One. 2019 Jun 26;14(6):e0218324. doi: 10.1371/journal.pone.0218324 (PMC6594600; doi:10.1371/journal.pone.0218324)
Supplement: S1 Appendix — (PDF) [file pone.0218324.s002.pdf]

S1 Appendix The method to calculate Andresen's S

The following describes the method used to calculate Andresen's S index [7–9].

The S index, and associated spatial point pattern test, is a method that can identify change at the local and global levels. It was originally developed by Andresen [7,8] and extended by Steenbeek and Wheeler [9,35]. The test compares the proportions of event types (crimes in this case) in each areal unit to identify whether there are statistically significant differences between them. The test can be directional (e.g. is the proportion of one event type greater than another?) or, as is the case here, binary (are the two event types similar or different?).

In the binary version of the test, the output is a global index of similarity, S_g, that ranges between 0 (no similarity) and 1 (perfect similarity) and is calculated as follows:

$$S_g = \frac{\sum_{k=0}^{C} S_l(k)}{C} \tag{4}$$

where C is the number of areal units and $S_l(k)$ is the similarity (0, dissimilar or 1, similar) for cell k. As $S_l(k) \in [0, 1]$ then $0 \leq S_g \leq 1$ where larger values ($S_g \to 1$) indicate greater similarity. As such, the S-Index measures the percentage of areas that share a similar spatial pattern. There are a number of versions of the test available with the most relevant being a full bootstrap, 'partial' bootstrap, and a proportion difference test. Here a proportional difference test is used identify statistically significant change using Fisher's exact test to (a chi-square test could also be used) at the local level.
